# Genetic-Based Optimization of 3D Burch–Schneider Cage With Functionally Graded Lattice Material

**DOI:** 10.3389/fbioe.2022.819005

**Published:** 2022-01-26

**Authors:** Manman Xu, Yan Zhang, Shuting Wang, Guozhang Jiang

**Affiliations:** ^1^ Key Laboratory of Metallurgical Equipment and Control Technology of Ministry of Education, Wuhan University of Science and Technology, Wuhan, China; ^2^ Hubei Key Laboratory of Mechanical Transmission and Manufacturing Engineering, Wuhan University of Science and Technology, Wuhan, China; ^3^ Research Center for Biomimetic Robot and Intelligent Measurement and Control, Wuhan University of Science and Technology, Wuhan, China; ^4^ School of Mechanical Science and Engineering, Huazhong University of Science and Technology, Wuhan, China

**Keywords:** genetic algorithm, bioengineering, lattice material, structural optimization, Burch-Schneider cage

## Abstract

A Burch–Schneider (BS) cage is a reinforcement device used in total hip arthroplasty (THA) revision surgeries to bridge areas of acetabular loss. There have been a variety of BS cages in the market, which are made of solid metal. However, significant differences in structural configuration and mechanical behavior between bone and metal implants cause bone resorption and interface loosening, and hence lead to failure of the implant in the long term. To address this issue, an optimal design framework for a cellular BS cage was investigated in this study by genetic algorithm and topology optimization, inspired by porous human bone with variable holes. In this optimization, a BS cage is constructed with functionally graded lattice material which gradually evolves to achieve better mechanical behavior by natural selection and natural genetics. Clinical constraints that allow adequate bone ingrowth and manufacturing constraint that ensures the realization of the optimized implant are considered simultaneously. A homogenization method is introduced to calculate effective mechanical properties of octet-truss lattice material in a given range of relative density. At last, comparison of the optimum lattice BS cage with a fully solid cage and a lattice cage with identical element density indicates the validity of the optimization design strategy proposed in this article.

## 1 Introduction

Genetic algorithm (GA) is an efficient way to solve optimization problems by mimicking the principle of natural evolution (Goldberg, 1989; Liu et al., 2021; Weng et al., 2021). In terms of naturally evolved structures, such as porous human bone, birds’ wing, and shell, GA could be an efficient method for bio-structure optimization. A BS cage is an acetabular reinforcement device used in THA revision surgeries for supporting bulk bone grafts (Schatzker et al., 1984). The current BS cage is made of solid metal, for example, titanium, stainless steel, and cobalt–chrome alloy, which has a stiffness of at least five to twenty times that of bone (Chang et al., 1990). Because of the great difference in the stiffness magnitude of prosthesis and bone, the orthopedic implant starts carrying most of the load while the bone lacks enough mechanical stimuli. The phenomenon of reduction in load was identified as stress shielding, followed by bone resorption, and, consequently, led to failure of the implant in the long term (Huiskes et al., 1992; Katoozian et al., 2001; Liu et al., 2021). Aiming at reducing the risk of stress shielding, flexible implants with relatively low stiffness are designed. However, high failure rates due to excessive stress along the bone–implant interface have been reported (Sun et al., 2020a). The bone–implant interface loosening, which results from micro-motion of the implant along its interface with bone, is another primary reason for the failure of the prosthesis. One result of the micro-motion is the decrease in the amount of bone that grows into the implant, hence leading to the instability of the implant. Several experiments on animals have been done to study the fixation of implants with porous surfaces by bone ingrowth, that is, biological fixation, which has been proven to be related to the pore size of the implant. Pore size in the range of 50–800 µm is typically studied and considered to provide adequate fixation strength (Bobyn et al., 1980; Bragdon et al., 2004; Sun et al., 2020b).

As the implant can be modified in quite a wide range, including geometry and mechanical properties, implant design has promise to address the bone resorption and bone–implant interface loosening problems. Studies on implant design with the goal of reducing the stress shielding phenomenon have been done extensively. Reducing the mass of the fully dense implant is one solution to reduce stiffness, subsequently reducing stress shielding; hence, shape and topology optimization are usually adopted. Gross considered the use of a hollow stemmed hip implant to reduce stress shielding and optimized the shape of the hollow in the stem while keeping the outer profile of the stem smooth (Gross and Abel, 2001). However, the strength of this hollowed structure with a large void area should be concerned. Ridzwan optimized the section of the femoral implant subjected to volume percentage range from 30% to 70% to reduce stress shielding (Ridzwan et al., 2006). As the density-based optimization method was adopted, the boundaries of optimum results are zigzag, and optimum results were represented by elements with relative density ranges from 0.89 to 1. Fraldi applied maximum stiffness topology optimization on the femur implant and obtained continuous element density distribution, ranging from 0 to 1, within the implant as a result of volume constraints (Fraldi et al., 2010; Chen et al., 2021b,a). However, he did not mention how to match these elements with various densities to certain microstructures in terms of effective material properties. Deng worked on the topology optimization of the cavity domain in the profile of the femur using the level set method, preserving the external shape of the original femur (Deng et al., 2016). In addition to reducing the mass of the solid material, that is, modifying the topology or geometry of the implant, another solution to reducing the stiffness of the implant is adopting light-weight materials, like composite material or cellular material. Analytical and numerical research on optimization of configuration of fiber-reinforcement composites in hip implants had been performed (Chang et al., 1990; Katoozian et al., 2001).

Due to the recent advances in additive manufacturing (AM) techniques, for example, selective laser melting (SLM) and electron beam melting (EBM), fabricating complex-shaped structures with cellular materials has become feasible (Jiang et al., 2021b). Light-weight cellular materials are heterogeneous and exhibit advanced mechanical and biological properties, which makes cellular materials attractive for bone implants in biological engineering (Jiang et al., 2021a; Yang et al., 2021). The properties of cellular materials are determined, on the one hand, by the constituent material they are made up from and, on the other hand, by the way the solid is distributed in the cell, that is, microstructural configuration. According to the microstructural configuration, cellular materials fall into two major categories: foam and lattice. Foam materials are made up of disordered voids with a variety of sizes which can produce reduced stiffness. In titanium-based foam, stiffness drops with the square of relative density, contributing to the reduction of stress shielding (Shen and Brinson, 2007; Luo et al., 2020). However, the porous microarchitecture of metallic foams causes stress and strain concentration near voids which reduces fatigue strength. Other than foam material, lattice material is structurally simple, which is built by replication of a periodic unit cell. Compared with foam, lattice material has relatively high strength. The competitive advantages of lattice material in optimization principally lie in two aspects: first, by using unit cells with given material properties, the cells can be used as mesh elements in numerical calculation, which could ease computational intensity of analysis and optimization; second, using lattice material to construct components provides direct control on the mechanical behavior of components at the macro-level by altering morphologic parameters of microstructures in the micro-level. Plenty of research had been done on the design of femoral stem implants with lattice displayed microstructure (Harrysson et al., 2008; Parthasarathy et al., 2011; Arabnejad Khanoki and Pasini, 2012; Arabnejad et al., 2017). In most work, the implant is built with a periodic microstructure with identical porosity of each cell, except the work Arabnejad had done. Arabnajad attempted to reduce stress shielding of two-dimensional femoral stem implants with hollow square unit cells.

By far, the bone implant design work mainly focused on the stress shielding phenomenon, and various methods had been proposed to address this problem. Besides, the femoral stem implant is the most studied implant due to its importance and its relatively simple geometry. To the best of our knowledge, no work has so far been done to optimize three-dimensional lattice BS cages with functional graded porosity, aiming at reducing bone resorption and improving bone–implant bounding strength concurrently.

As human bone with variable pores is the result of long-term genetic evolution, this study proposes an optimization framework for cellular BS cages by genetic algorithm. In this method, the BS cage is featured with lattice material with varied densities, and each cell of the lattice is viewed as a chromosome while a crossover or mutation operator is used to create new generations. Consequently, the optimized BS cage has variable pore sizes like human bone and hence performs applicable biomechanical properties and provides biological fixation. Besides, manufacturing constraint that considers the minimum strut size is guaranteed for the fabrication of the designed implant.

## 2 Design Methods

### 2.1 Biological Problem Statement

**FIGURE 1:**
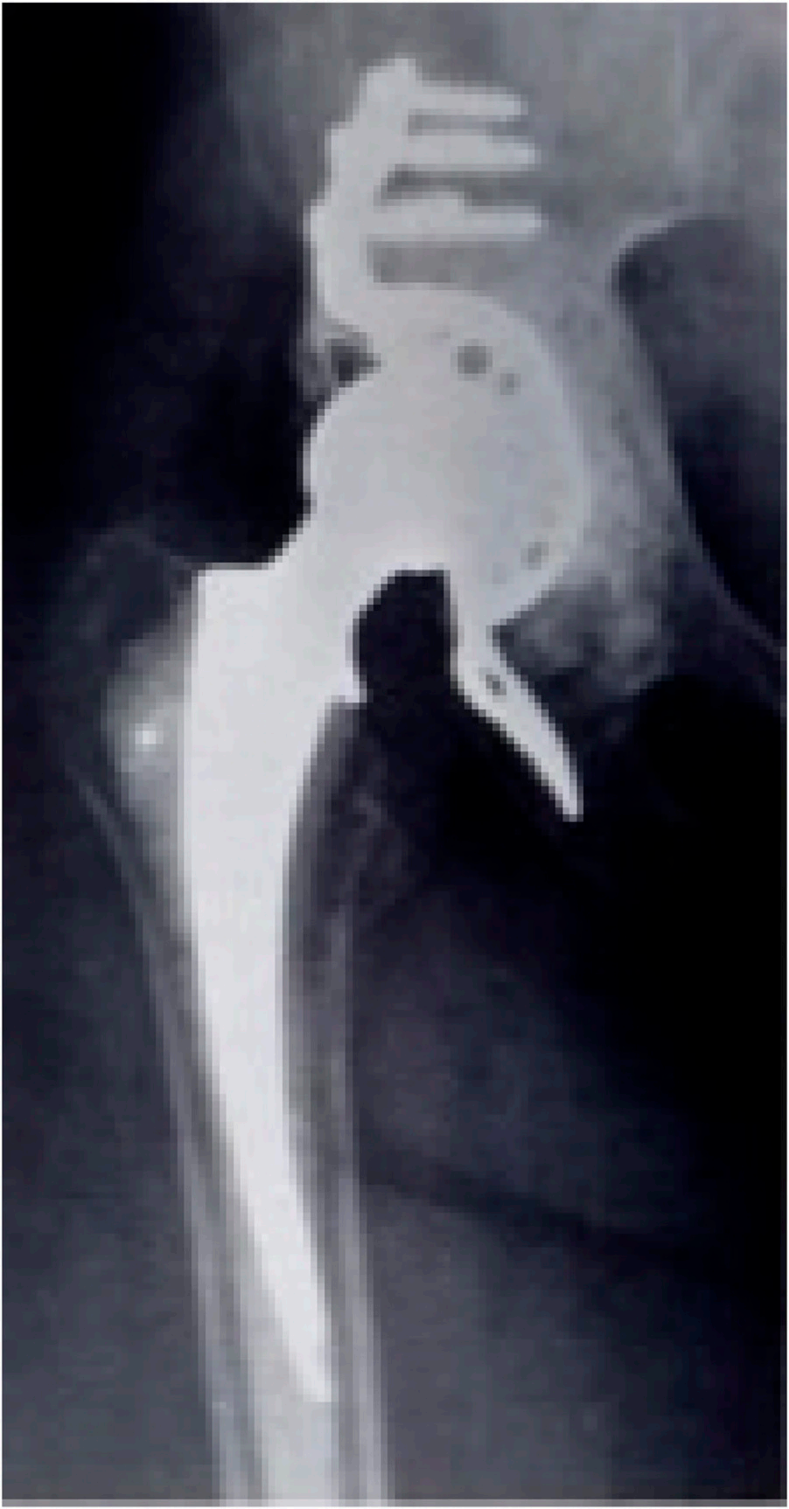
Radiograph of a patient’s right hip with a BS cage (Rosson and Schatzker, 1992).

The BS cage was first developed by Dr. Burch for the treatment of a patient with old, unhealed acetabular fracture in 1974, and Dr. Schneider developed it further in 1975 (Hoell et al., 2012). Figure 1 is a radiograph of a patient’s right hip 3 years after reconstructing with a BS cage. A BS cage consists of a hemispherical shell, an inferior flange inserted into a prepared slot in the ischial bone to ensure rotational stability, and a superior flange resting against the ilium. Besides, three to five screws are inserted into the pelvis bone to supply fixation. Owing to its geometry, a BS cage can provide a large contact between the implant and the pelvis bone, distributing the joint force over a large area, hence achieving long-term stability. The BS cage has been recognized as the first implant that could reconstruct massive acetabular defect due to its high support on the acetabular socket and the femur stem.

After the fixation of the BS cage, a polyethylene acetabular liner is cemented to the socket of the BS cage, which is used to orientate the femoral head. The femoral head is generally re-established bone-replacement implant, as fracture on acetabular occurs simultaneously with defect on the femoral head it connected with.

Accurate geometry models, materials, and boundary conditions are key to analyzing the mechanical behavior of the BS cage and the pelvis bone. Due to the complexity of the pelvis structure, the pelvis model is constructed by 297 CT scans of a male weighing 80 kg in this study. In order to capture precise details of the BS cage, 3D scan is introduced to obtain points on the surface of the BS cage; the point clouds are subsequently used to create a CAD model. A BS cage is made from titanium as titanium has adequate cost and stiffness compared to other biomaterials, for example, tantalum and stainless steel. Titanium is also used in the femoral stem implant, with a Young’s modulus of 110 GPa and a Poisson’s ratio of 0.3. Material properties of porous BS cages with lattice material will be introduced in detail in the next section. The liner between the femoral stem and the BS cage is made from polyethylene, with a Young’s modulus of 1 GPa and a Poisson’s ratio of 0.4. There is cement between the liner and the BS cage for fixation of the liner, and the Young’s modulus of cement is 2.5 GPa and the Poisson’s ratio of cement is 0.3. The Young’s modulus and Poisson’s ratio vary in different parts of the pelvis bone; hence, the pelvis is heterogeneous.

In many previous research studies, a rough description of material properties of the pelvis bone is adopted by assuming the pelvis is a two-phase structure (Manley et al., 2006; Phillips et al., 2007; Plessers and Mau, 2016). In these studies, the pelvis bone is simplified as a structure with a thin cortical outer shell and a trabecular inner region. However, it has been found that the strain of the cortical bone is very sensitive to cortical thickness (Anderson et al., 2005). Hence, a finite element (FE) pelvis model with accurate description of material plays an important role in predicting mechanical response. In this study, the relationship between material properties of the pelvis and the gray value of its CT scan images is built to estimate the heterogeneous material model of the pelvis. It has been demonstrated that the density of bone tissue is linear to the gray value, Hounsfield Unit (HU), of pixel in CT images. The material properties of bone have a power relationship with the density of bone tissues. Thus, HU can be used to estimate the material properties of the pelvis bone (Baca and Horak, 2007). The Young’s modulus is formulated as follows:

$$E=\left\{\begin{array}{cc} 17000\;MPa\; & whenHU⩾1500\\ 2713\rho_{0}^{2.36}\;MPa\; & \;when100⩽HU⩽1500\\ 20\;MPa\; & whenHU⩽100 \end{array}\right.$$ (1)

in which,

$$\rho_{0}=\frac{HU}{1500}\cdot 2.0\;{g/cm}^{3}$$ (2)

**FIGURE 2:**
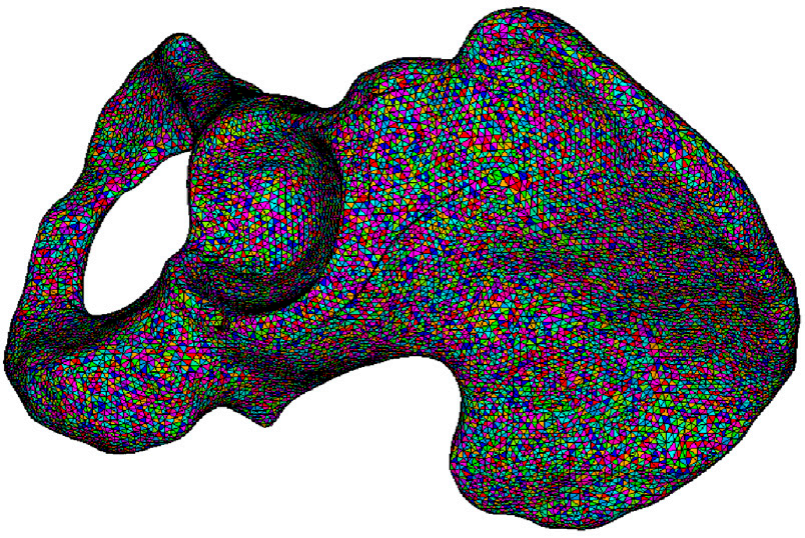
Mesh model of the human left pelvis with heterogeneous material.

As defined in Eq. 1, the pelvis bone is divided into three types: cortical bone, cancellous bone, and bone marrow, corresponding to HU⩾1500, 100⩽HU⩽1500, and HU⩽100, respectively. According to the classification of bone, the relationship of Young’s modulus and HU is represented by a piecewise function with each HU corresponding to a specific Young’s modulus. For cancellous bone, its density is derived with regard to the HU it associated with and the density of cortical bone, which is assumed as 2.0 g/cm^3^. The Poisson’s ratio of the pelvis bone is assigned as 0.3. By mapping each pixel of CT scan images to elements in the FE model of the pelvis, elements are assigned to material properties calculated by the HU of corresponding pixel (Korshunova et al., 2021). The mesh model of the pelvis bone is depicted in Figure 2, in which the pelvis is built with heterogeneous material and each material is marked with one color.

**FIGURE 3:**
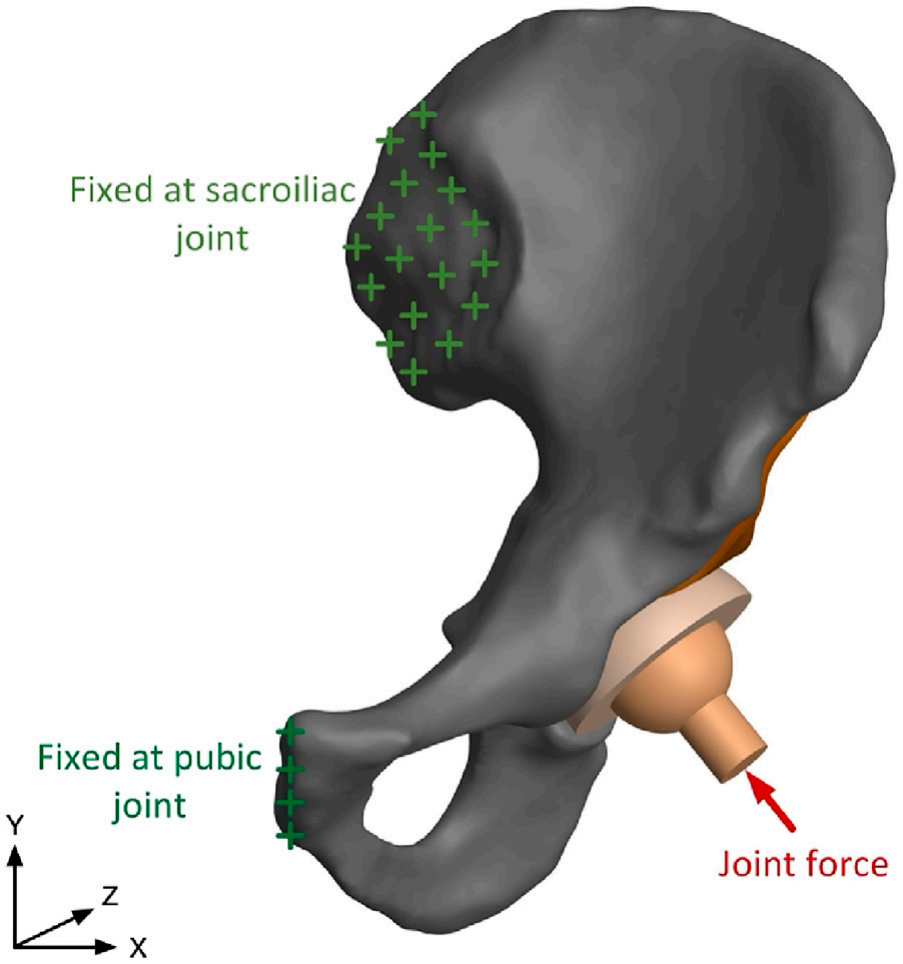
Assembly model of the human left pelvis and boundary conditions.

After the generation of the FE model with assignment of material properties, boundary conditions are applied as depicted in Figure 3. The human pelvis is constrained by joint and ligaments, which could be simplified as two major components: the pubic symphysis and the sacroiliac joint (Hao et al., 2011). In previous research, contact force in the hip joint has been tested, and average force of walking and standing on one leg is 238% of body weight, and force is 251% of body weight when climbing upstairs. In this study, resultant force in the hip joint is 1962N, 250% of body weight, which is -408N in X direction and 1919N in Y direction (Bergmann et al., 2001). Numerical calculation of the assembly model is conducted in ANSYS, where solid 186 elements are used for the cage and solid 185 elements are used for other parts.

### 2.2 Mechanical Properties of Lattice Materials

#### 2.2.1 Homogenization Method

The homogenization method has been recognized as a rigorous method for calculating effective properties of lattice material or composite materials characterized by periodic microarchitectures. Microstructure is the smallest repeating cell that represents the overall configuration and properties of bulk materials; hence, one microarchitecture is treated as a representative volume element (RVE) in numerical analysis (Hassani and Hinton, 1998). Structures made of these materials are considered heterogeneous at the microscopic level, while being viewed as homogeneous at the macroscopic level. The main idea of homogenization is finding a homogenized material constant to describe the behavior of heterogeneous material, of which quantities vary slowly on macroscopic while quantities oscillate rapidly on microscopic, by asymptotic expansion in the two scales (Auricchio et al., 2020; Carraturo et al., 2021; Deng and To, 2021). Taking the quantities on the microscale into consideration, field quantities on the macroscale can be expanded as follows:

$$\Phi^{\varepsilon }\left(x\right)=\Phi^{0}\left(x,y\right)+\epsilon \Phi^{1}\left(x,y\right)+\epsilon^{2}\Phi^{2}\left(x,y\right)+\cdots$$ (3)

where *x* and *y* are the real length of the unit vector in microscopic and macroscopic coordinates, respectively, and *ϵ* = *x*/*y* has a small value. The superscript *ϵ* of quantities is an indication of the double scale. For the linear elasticity problem, displacement and stress are quantities that need to be asymptotically expanded in the homogenization method, and the relation of displacement and stress follows Hook’s law, that is, the equilibrium equation:

$$\sigma_{ij}^{\epsilon }=E_{ijkl}^{\text{H}}\varepsilon_{kl}^{\epsilon }$$ (4)

$$\varepsilon_{kl}^{\epsilon }=\frac{1}{2}\left(\frac{\partial u_{k}^{\epsilon }}{\partial x_{l}}+\frac{\partial u_{l}^{\epsilon }}{\partial x_{k}}\right)$$ (5)

$\sigma_{ij}^{\epsilon }$ is the stress, $\varepsilon_{kl}^{\epsilon }$ is the strain, $u_{k}^{\epsilon }$ and $u_{l}^{\epsilon }$ are displacements, and $E_{ijkl}^{\text{H}}$ is the effective elastic tensor which can be derived as follows:

$$E_{ijkl}^{\text{H}}=\frac{1}{\left|Y\right|}\int_{Y^{s}}\left(\varepsilon_{pq}^{0}-\varepsilon_{pq}\right)E_{pqrs}\left(\varepsilon_{rs}^{0}-\varepsilon_{rs}\right)dY$$ (6)

where $\left|Y\right|$ is the volume (area) of the entire RVE, *Y* ^*s*^ is the solid in the RVE, *E*_*pqrs*_ is the elastic matrix of the solid material the structure is made from, $\varepsilon_{pq}^{0}$ , $\varepsilon_{rs}^{0}$ are unit test strain applied on the boundary of the RVE, and *ɛ*_*pq*_ and *ɛ*_*rs*_ are the corresponding induced strain. For the three-dimensional structure, the elastic matrix is a 6 × 6 matrix, which can be calculated by applying the test strain six times; each time, one strain component was set to the unit whereas the remaining five are zero.

Once the effective elastic matrix is obtained, the effective compliance matrix is calculated as follows:

$$C_{ijkl}^{\text{H}}={E_{ijkl}^{\text{H}}}^{-1}$$ (7)

where the compliance matrix $C_{ijkl}^{\text{H}}$ is a matrix in terms of elastic constants, and for orthotropic material, the compliance matrix can be written as follows:

$$C_{ijkl}^{\text{H}}=\left[\begin{array}{cccccc} \frac{1}{E_{1}} & \frac{-\nu_{12}}{E_{1}} & \frac{-\nu_{13}}{E_{1}} & 0 & 0 & 0\\ & \frac{1}{E_{2}} & \frac{-\nu_{23}}{E_{2}} & 0 & 0 & 0\\ & & \frac{1}{E_{3}} & 0 & 0 & 0\\ & symmetric & & \frac{1}{G_{12}} & 0 & 0\\ & & & & \frac{1}{G_{13}} & 0\\ & & & & & \frac{1}{G_{23}} \end{array}\right]$$ (8)

Hence, the effective Young’s modulus, Poisson’s ratio, and shear modulus of heterogeneous lattice material are obtained.

#### 2.2.2 Mechanical Properties of Lattice Truss Materials

Lattice material is typically composed of spatially periodic unit cells which are specified by structural elements, such as rods and beams, interconnected at nodes. Due to the topology similarity of unit cells with macro truss structures, this type of lattice material is also named lattice truss material. The struts mainly express uniaxial tensile or compressive deformation when subject to macro load; hence, lattice truss material is always stretching dominated. Tension and compression properties are more important indications to a BS cage, which is a thin shell structure, other than shear behavior. Mechanical properties of lattice truss material have been studied analytically and experimentally in previous work, where each strut is assumed to be pinned at its corresponding node under the precondition that the lattice has low relative density (Fan et al., 2008). In these works, Young’s modulus is the property that the study mostly focused on, and analytical Young’s modulus is expressed as a linear equation in terms of relative density. Although the properties of lattice truss materials have been analyzed, numerical studies on the properties of three-dimensional lattice truss material are lacking.

**FIGURE 4:**
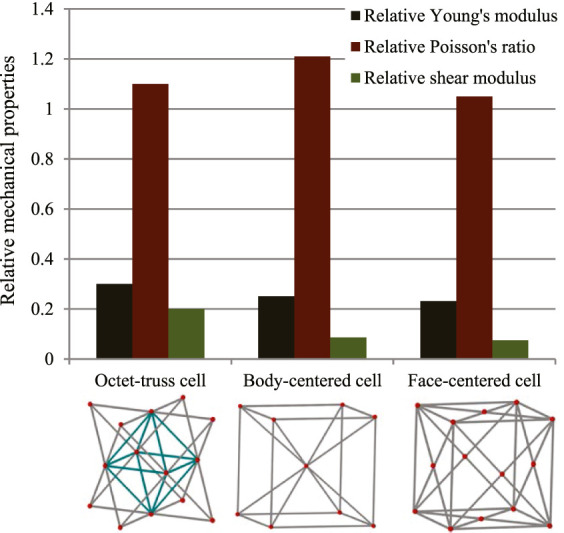
Relative effective mechanical properties of three typical stretching-dominated unit cells.

**FIGURE 5:**
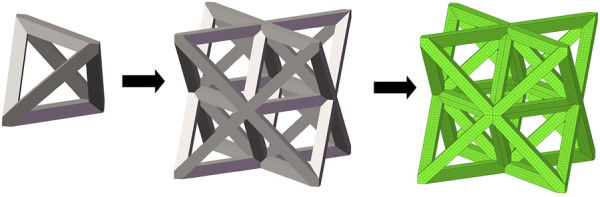
Geometry and mesh model of one octet-truss unit cell.

**FIGURE 6:**
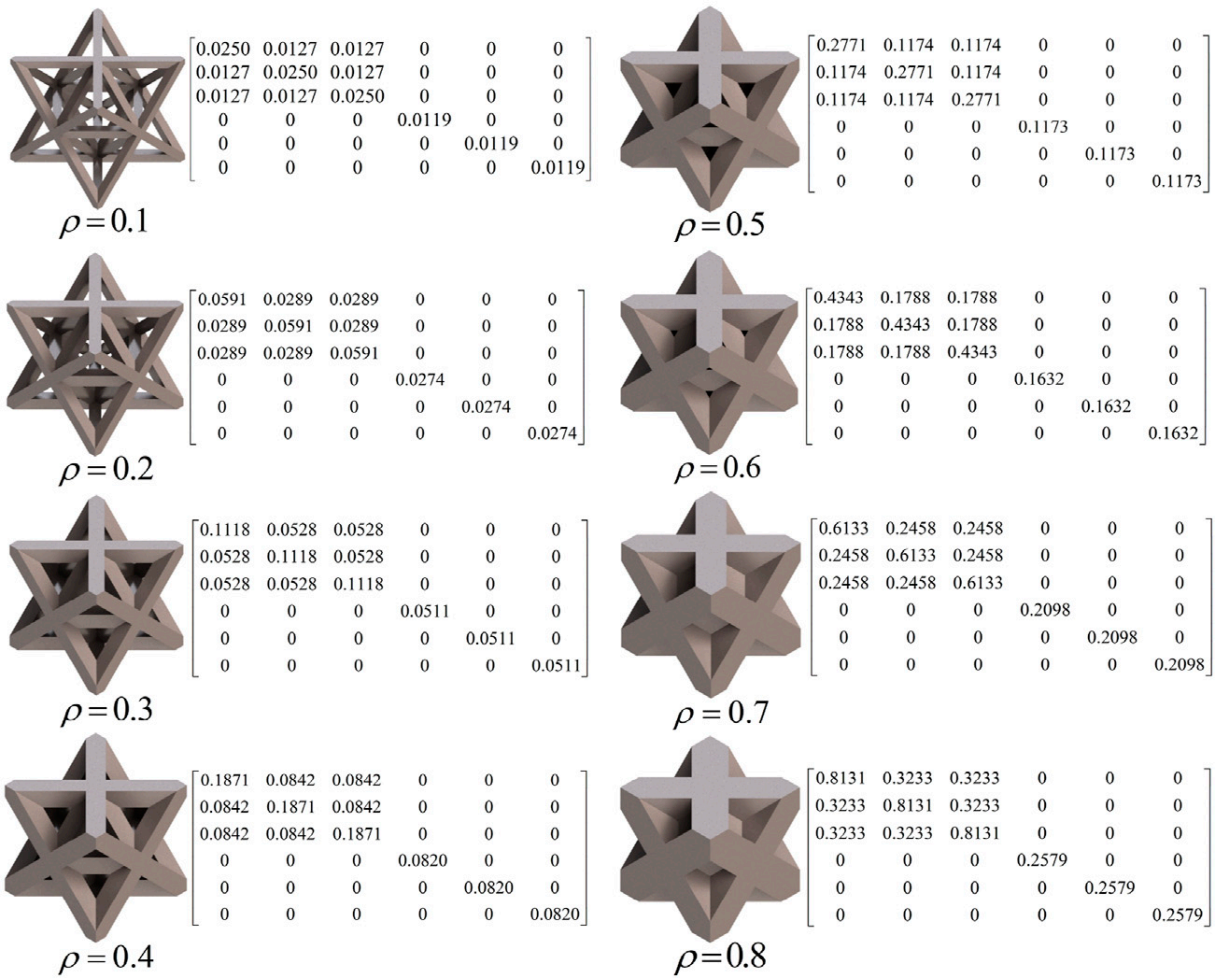
Elastic matrix of an octet-truss cell with relative density ranging from 0.1 to 0.8.

In this study, three typical unit cells of stretching dominated lattice are selected and the homogenization method is used to calculate the effective material properties of corresponding lattice materials. Structural configuration of these unit cells, that is, octet-truss, body-centered, face-centered, and effective material properties of corresponding lattice material with relative density of 0.5 are depicted in Figure 4. Relative material properties are ratios of material constants of lattice material to that of solid materials the lattice is made up from. Due to geometrical symmetry of these three cells, the values of elastic constants in three directions are equal, and constant in one direction is exhibited in figures and hereafter for brevity. From Figure 4, it is observed that the octet-truss cell is composed of one octahedral with 6 nodes and 12 rods and four tetrahedral cells. Octet-truss has relatively higher Young’s and shear modulus than body-centered and face-centered cells that could reduce tensile and shear deformation during use. Poisson’s ratio of octet-truss is medium to reduce transverse deformation. Octet-truss has the best performance in resisting deformation on the whole; hence, the octet-truss cell is chosen as the RVE of the lattice BS cage to improve the strength of this shell structure. The geometry and mesh model of one octet-truss cell is depicted in Figure 5, in which the whole geometry and mesh model is built from a 1/8 octet-truss due to the geometry symmetry. Effective properties of octet-truss cells with a relative density ranging from 0.1 to 0.8 are calculated using the homogenization method for optimization, and the elastic matrices are shown in Figure 6.

**FIGURE 7:**
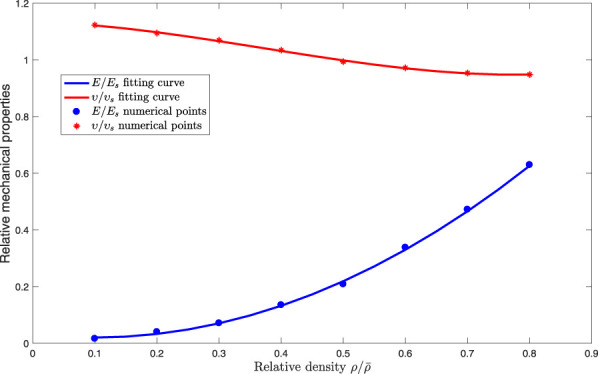
Fitting function curves and discrete numerical points of effective properties with respect to relative density of octet-truss cells.

The relation between relative element density and effective mechanical properties of octet-truss lattice is precisely obtained. Continuous expression of effective mechanical properties with respect to relative element density is beneficial to reducing computational cost in optimization. Hence, a continuous function of effective mechanical properties with respect to relative density of cells is introduced in this case. According to the effective mechanical properties calculated previously, a continuous effective material property function with respect to element density is achieved by polynomial fitting functions:

$$\bar{E}=\left(1.23\rho_{e}^{2}-0.2411\rho_{e}+0.03213\right)E_{s}$$ (9)

$$\bar{\upsilon }=\left(0.7165\rho_{e}^{3}-0.7995\rho_{e}^{2}-0.05101\rho_{e}\;+1.134\right)\upsilon_{s}$$ (10)

where $\bar{\upsilon }$ and $\bar{E}$ are effective Poisson’s ratio and Young’s modulus, and *υ*_*s*_ and *E*_*s*_ are Poisson’s ratio and Young’s modulus of solid material, which is titanium in this case. The degree of accuracy of these fitting functions scaled by R-square is above 0.998, and the fitting function curve, together with discrete points, is shown in Figure 7. In Figure 7, the *y* axis represents the ratio of effective properties of lattice material composed of octet-truss cells to properties of fully solid material the lattice is made up from. It can be seen that effective Young’s modulus is approximately linear to relative density at very low density, which is in agreement with analytical results in previous research.

### 2.3 Optimization of Functionally Graded Cellular BS Cage

Cellular bone implants made up of lattice material provide an exciting opportunity for orthopedic applications and mechanical properties of lattice implants. Lattice implants should ideally be tailored to match the stiffness of the host bone so as to reduce bone resorption induced by stress shielding. Mechanical properties of lattice implants can be altered by varying the topology of unit cells; hence, the mechanical response of the implant structure at the macroscale level can be characterized by control on the topology of RVEs. Optimization of a functionally graded cellular BS cage turns into an optimization of topology of RVEs of lattice material the BS cage is made up from.

In this case, the topology of the given octet-truss cell is characterized by its morphological parameters, for example, strut thickness, pore size, cell size, and porosity. By geometric analysis, the relation between porosity and strut thickness is built as follows:

$$p=1-\rho =1-6\sqrt{2}\pi {\left(\frac{t}{l}\right)}^{2}$$ (11)

where *p* is porosity of the cell, measured as percentage of the void in a fully solid cell, *ρ* is the density of the cell, *t* is strut thickness, and *l* is strut length. Any morphological parameter of a cell can be calculated by the other two morphological parameters.

In optimization, specific constraints related to practical application other than volume constraint of components should be taken into consideration. The BS cage is fabricated by AM, which has a limitation on the resolution of the lattice struts: a minimum strut thickness of 200 µm is required to ensure accuracy in the dimensions of the manufactured parts. Besides, it has been demonstrated that the pore size between 50 and 800 µm allows proper bone ingrowth and secondary long-term biological stability.

**FIGURE 8:**
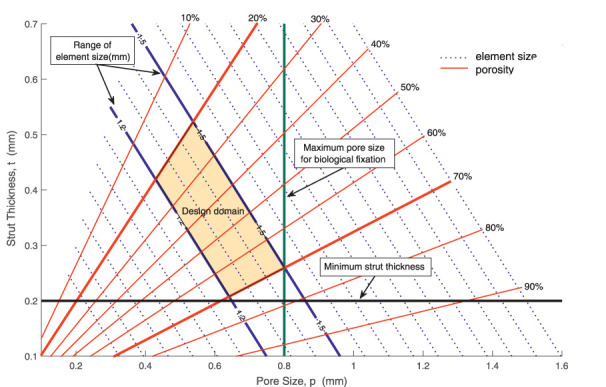
Interplay between morphological parameters of an octet-truss cell and design constraints.

The interaction between morphological parameters, together with clinical and manufacturing constraints, is described in Figure 8. In Figure 8, pore size *p* and strut thickness *t* are set as the horizontal and vertical axis, respectively. As described in Eq. 11, element size *l* and porosity *ρ* can be calculated by pore size and strut thickness, and a series of contour lines of element size and porosity are illustrated in Figure 8. The size of elements used in this work is depicted by solid blue lines, which ranges from 1.2 to 1.5 mm. Besides, two essential constraints, that is, manufacturing and biological constraints, are considered. A minimum 3D manufacturing strut thickness of 0.2 mm is represented by the black line, while a maximum pore size of 0.8 mm for biological fixation is represented by the green line. Taking into consideration both clinical constraints and manufacturing constraints, the admissible design space is highlighted in orange color. Any point in this domain is feasible to meet biological fixation requirement and manufacture limitation. According to the design domain, porosity of RVEs is set as design variables, and the value of porosity is limited from 0.2 to 0.7 with given RVE size.

The optimization objective of the BS cage is minimization of compliance, which could reduce stress concentration by redistributing materials appropriately and enhance the strength to support loading from the hip joint. Based on the morphological parameters analyzed earlier, the optimization is formulated as follows:

$$\begin{array}{cc} Minimize:\; & c\left(\rho \right)=U^{\text{T}}KU\\ Subjectto:\; & KU=F\\ & V\left(\rho \right)\leq V_{0}\\ & 0.2\leq \rho \leq 0.7 \end{array}$$ (12)

where *c* is the compliance, **U** is the global displacement matrix, **K** is the global stiffness matrix, **F** is the force vector. V is the material volume of the component, *ρ* is the element density, and *V*_0_ is the volume constraint which is set as 0.4 in this study to facilitate the bone ingrowth.

In the optimization process, each element density is coded as a binary string and the fitness value is the elemental sensitivity which is equal to the element strain energy as follows:

$$\Delta c_{e}=u_{e}^{\text{T}}{\bar{k}}_{e}u_{e}$$ (13)

where **u**_*e*_ is the nodal displacement vector of the element, and **k**_*e*_ is the element stiffness matrix, which is related to element density *ρ*_*e*_. The initial values of element densities are set as the same value which equals to volume constraint 0.4. Based on the fitness values, choice probabilities of the father and mother gene are determined for crossover procedure. Mutation of the density gene allows the element with high fitness value to evolve to high density and the element with low fitness value to evolve to low density. In this optimization, the crossover factor is set as 0.3 and the mutation factor is set as 0.2. After crossover and mutation, each element individual of the population obtains a new gene. The optimization procedure is terminated when the objective values between successive iterations is less than 10^–3^.

## 3 Results

**FIGURE 9:**
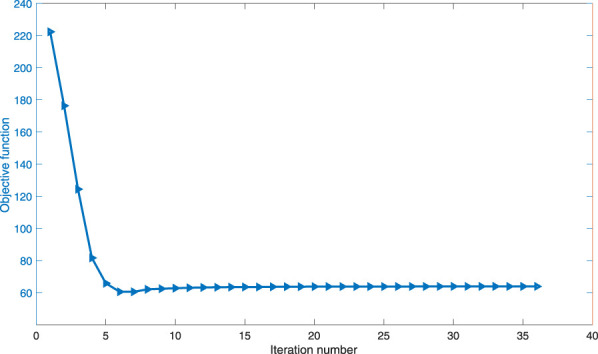
Convergence history of the objective in optimization of the BS cage.

In the optimization process, the convergent history of the objective is shown in Figure 9. The compliance of the cage varies until the 36th iteration, when the convergence criterion is satisfied and hence iteration is terminated.

**FIGURE 10:**
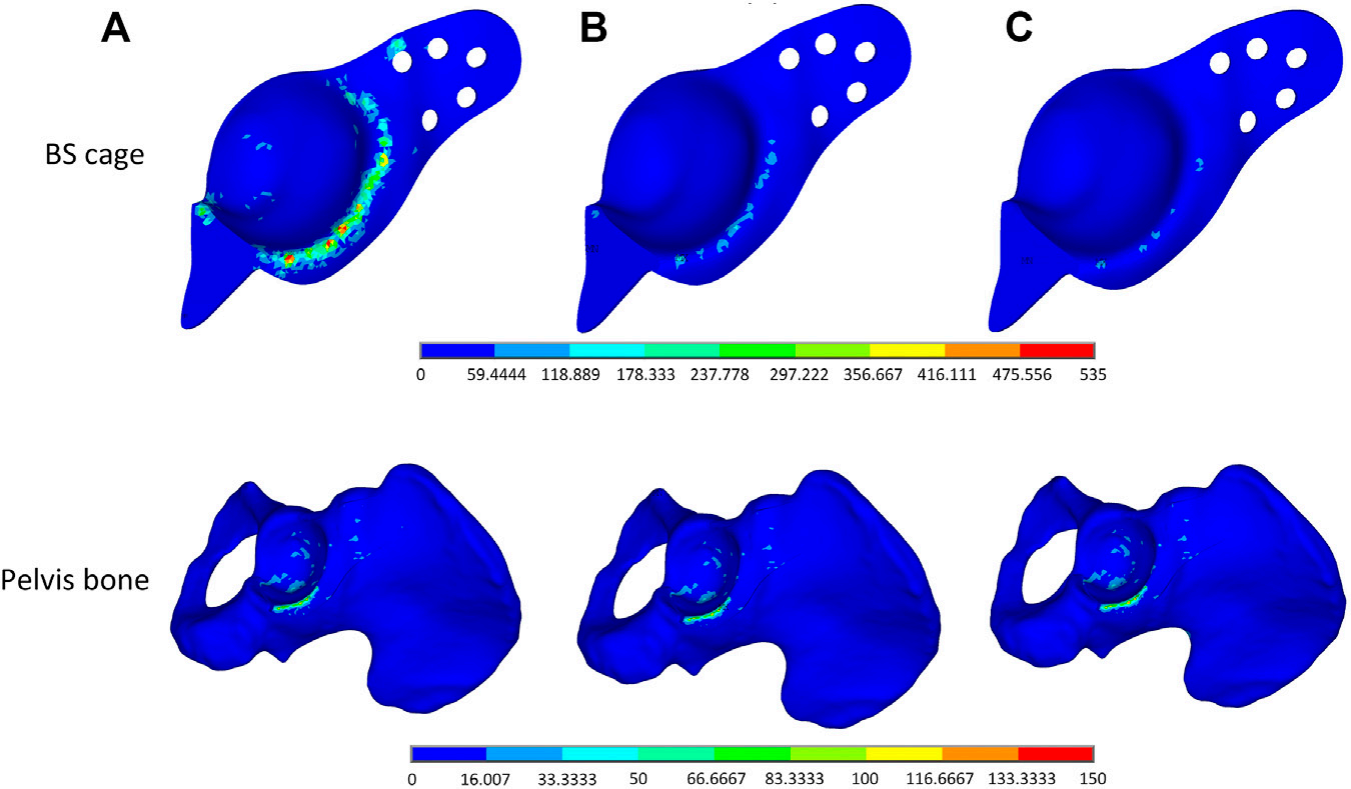
Von-Mises stress distribution plot of **(A)** a full solid BS cage and the corresponding pelvis bone, **(B)** a lattice BS cage with an identical element density of 0.4, and **(C)** a lattice BS cage with an optimized graded element density under volume constraint of 0.4.

**FIGURE 11:**
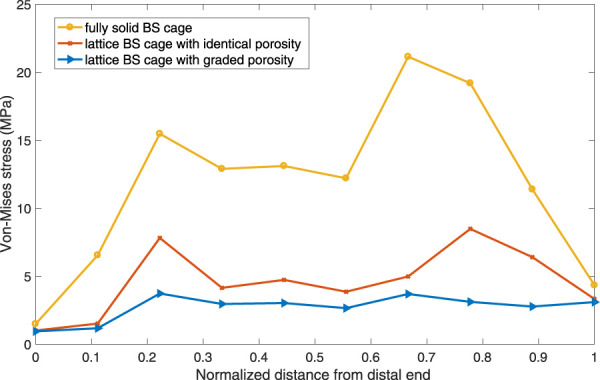
Von-Mises stress distribution at the bone–implant interface.

To verify the validity of the optimization performed on the BS cage, a comparison of performance of the BS cage and the pelvis bone it attached in three different cases is carried out. In the cases, three different BS cages, which are a fully metal cage, a porous cage with identical porosity, and a porous cage with optimized graded porosity, are implemented, and the Von-Mises stresses distribution of both BS cage and pelvis are shown in Figure 10. The Von-Mises stresses are highest in the fully solid cage (a) and lowest in the functionally graded lattice cage with tailored mechanical properties after optimization (c). As can be seen from Figure 10, there is not obvious variation in the stresses of the pelvis bone in the three cases. However, the values of maximum Von-Mises stress of the pelvis bone in case (c) is lower than it is in the other two cases. For the comparison of stresses over the bone–implant interface, several points from the distal end to the proximal end of the cage are selected, and stress values on these points are illustrated in Figure 11. It indicates a substantial reduction in the interface stress of the optimum lattice cage with respect to the fully solid cage, which helps to reduce the risk of interface debonding.

The maximum Von-Mises stress of the lattice cage with optimum porosity is 135.835 MPa, which is about 18% less than that of the lattice cage with an identical porosity of 0.5 and 75% less than that of the fully metal cage. The fatigue of pure titanium for 10^7^ cycles is about 425–700 MPa, which varies depending on the precise material characteristics and the production process (Pazos et al., 2010; Figueiredo et al., 2014). As the maximum Von-Mises stress of the optimized lattice BS cage is much less than its fatigue stress, the mechanical behavior of the optimized cage is considered guaranteed.

**FIGURE 12:**
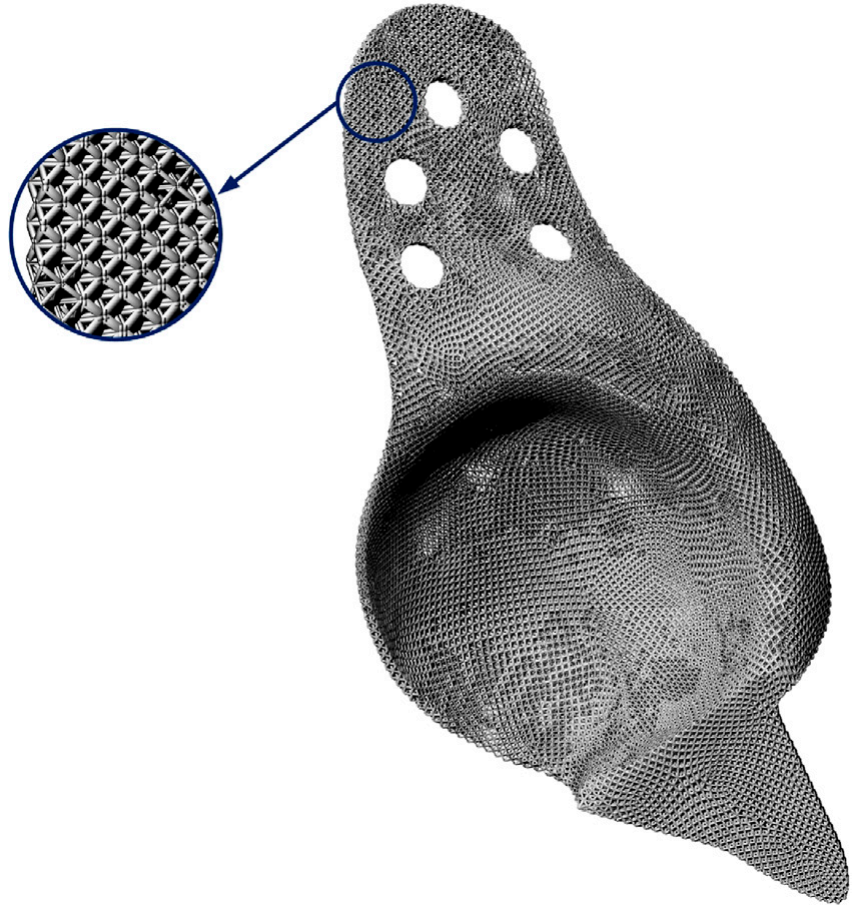
Final CAD model of the functionally graded lattice BS cage and its partial enlarged view.

After obtaining the cage model with graded element densities, a post-processing is essential to mapping the mesh model with characterized element density to a CAD model with lattice material, where each unit cell of lattice corresponds to a mesh element in the mesh model of the cage. Hence, a Python code is developed to realize the mapping process and build the CAD model of the optimized lattice cage model with graded porosity of element in Rhinoceros. The final model of the optimum lattice BS cage model is shown in Figure 12.

## 4 Conclusion

In this study, a functionally graded porous orthopedic prosthesis made up of lattice material is designed based on genetic algorithm. The BS cage, used in THA revision surgeries to bridge areas of acetabular loss and provide support to the acetabular socket, is the optimization object in this study. In the optimization design, lattice material constructed by replication of periodic unit cells is introduced due to its adequate mechanical properties, relatively low stiffness to reduce stress shielding, and biomechanical properties; it possesses pores for bone ingrowth. Besides, lattice material allows for direct control on mechanical properties of components made up by it. After analysis and comparison, octet-truss lattice is selected in this study and mechanical response of octet-truss lattice material with cell density in a given range is calculated using the homogenization method. Taking into consideration both manufacturing constraint and clinical constraint, interplay between morphologic parameters of the octet-truss cell is obtained; subsequently, design constraints are determined. Therefore, this scientific problem of designing a graded density porous BS cage with tailored mechanical properties is formulated as an optimization problem for the relative density of elements of a BS cage with the goal of maximizing the structural compliance. Finally, a BS cage with functionally graded lattice material is achieved by optimization, which allows biological binding with the pelvis bone and reduces stress on the BS cage and the pelvis bone. A full solid cage and a lattice cage without optimization are set as references to verify the effectiveness of the design framework. This paper can contribute to the practical applications of topology optimization in bioengineering. An octet-truss unit cell has good mechanical property compared with other unit cells. However, a single unit cell with prescribed structural configuration limits the design space of this optimization problem. In future work, several unit cells could be used simultaneously to improve the performance of the porous BS cage.

## Data Availability

The raw data supporting the conclusions of this article will be made available by the authors, without undue reservation.
